# Smokers’ Use of E-Cigarettes in Situations Where Smoking Is not Permitted in England: Quarterly Trends 2011–2020 and Associations With Sociodemographic and Smoking Characteristics

**DOI:** 10.1093/ntr/ntab119

**Published:** 2021-06-05

**Authors:** Sarah E Jackson, Emma Beard, Jamie Brown

**Affiliations:** 1 Department of Behavioural Science and Health, University College London, London, UK; 2 SPECTRUM Consortium, UK

## Abstract

**Introduction:**

This study aimed to examine how the proportion of dual users of cigarettes and e-cigarettes who use e-cigarettes in situations where smoking is not permitted has changed since e-cigarettes became popular in England, and to characterize those who do so.

**Methods:**

Data were from 5081 adults in England who reported current smoking and current use of e-cigarettes (‘dual users’) participating in a nationally-representative monthly survey between April 2011 and February 2020. We modeled quarterly changes in prevalence of e-cigarette use in situations where smoking is not permitted and assessed multivariable associations with sociodemographic and smoking characteristics.

**Results:**

Between 2011 and 2020, prevalence of e-cigarette use in situations where smoking is not permitted followed a positive cubic trend, with a decelerating increase from an estimated 52.5% of dual users in Q2-2011 to 72.7% in Q3-2014, followed by a small decline to 67.5% in 2018, and subsequent increase to 74.0% in 2020. Odds were higher among those who were from more disadvantaged social grades, reported stronger smoking urges, or had made a past-year quit attempt, and lower among those who were aged at least 65 years (vs. 16–24 years), from the south (vs. north) of England, reported currently cutting down on their cigarette consumption or currently using nicotine replacement therapy.

**Conclusions:**

In England, use of e-cigarettes in situations where smoking is not permitted is common among dual cigarette and e-cigarette users, has increased nonlinearly since 2011, and is particularly prevalent among those who are younger, disadvantaged, more addicted, have recently failed to quit, and are not attempting to cut down.

**Implications:**

The ability to use nicotine in smoke-free settings appears to be an important part of the appeal of e-cigarettes. It is possible that if e-cigarette use was prohibited in public places, smokers may be deterred from using e-cigarettes alongside combustible tobacco, which could undermine quitting. Our results suggest disadvantaged and more addicted smokers would be disproportionately affected, suggesting such restrictions may contribute to inequalities in smoking and associated health outcomes.

## Introduction

E-cigarettes have rapidly become popular among smokers over the last decade. In England, the prevalence of e-cigarette use (“vaping”) increased tenfold between 2011 and 2014, from 2% to 21%.^1^ Since then, it has plateaued and remained relatively stable at around 20%.^1^ Meanwhile, cigarette smoking prevalence has declined steadily from 20% in 2011 to 14% in 2019.^2^ While cessation and harm reduction are the most commonly reported reasons for vaping,^3–7^ the ability to use nicotine in situations where smoking is not permitted seems to be another important part of e-cigarettes’ appeal.^3,4,6^ Determining regulation to optimize the public health impact of e-cigarettes is a global priority, including how to approach the use of e-cigarettes in smoke-free and public places. There have been calls for a wider debate on how e-cigarettes are to be dealt with in public places.^8^ Understanding population trends in the use of e-cigarettes to achieve temporary abstinence from smoking in situations where smoking is not permitted can inform this debate and allow judgments on the number of people likely affected.

In 2018, comprehensive smoke-free legislation covered approximately 1.6 billion people in 62 countries, rising from 0.2 billion people in 10 countries in 2007.^9^ In England, legislation banning tobacco smoking in enclosed (or substantially enclosed) public places (including public vehicles) and workplaces was brought into effect in 2007. In 2008, the ban was extended to Mental Health Units and to private vehicles in 2015. While these laws do not cover vaping, an increasing number of businesses and public transport operators now treat vaping in the same way as smoking, applying a blanket ban that prohibits both smoking and the use of e-cigarettes. This is in spite of consensus across England’s public health community that e-cigarettes are substantially safer than smoked tobacco, a lack of evidence of harm to bystanders from exposure to e-cigarette vapor,^5^ and guidance issued by Public Health England that encourages evidence-based policies that distinguish between smoking and vaping.^10^ In this context, extension of smoking bans to e-cigarettes appears to be driven by concerns than some bystanders find exposure to e-cigarette vapor unpleasant.^8^ At the discretion of nongovernmental organizations and businesses across England, the extension of smoke-free policies to include use of e-cigarettes could at least partly explain the plateau in e-cigarette use over recent years.

It has been suggested that use of e-cigarettes as an alternative to smoking in smoke-free settings may promote quitting among smokers not currently planning to stop.^5^ Indeed, use of nicotine replacement therapy (NRT) for temporary abstinence has previously been shown to be associated with subsequent quit attempts and cessation.^11^ With e-cigarettes since overtaking NRT to become the most popular noncombustible nicotine product used by smokers in England,^12^ the potential for use of e-cigarettes for temporary abstinence to increase rates of quitting is significant. However, if smoking bans have been widely extended to include e-cigarettes this may make vaping less attractive and undermine this potential pathway to quitting via e-cigarettes. On the other hand, it is possible that the ability to use e-cigarettes in places where smoking is not permitted may prolong smoking behaviors rather than facilitate smoking cessation by enabling nicotine use in locations where this was not previously an option. Understanding how motives for e-cigarette use are changing over time is important for evaluating current policy about restrictions on e-cigarette use in public places and the extent to which it may affect smoking and quitting behavior.

The primary aim of this study was to examine whether, and if so to what extent, the proportion of smokers who use e-cigarettes in situations where smoking is not permitted (ie, for temporary abstinence) has changed since e-cigarettes first became popular in England. The secondary aim was to characterize smokers who use e-cigarettes in situations where smoking is not permitted. Specifically, we addressed the following research questions:

1. What function best characterizes the trend in use of e-cigarettes by dual users of cigarettes and e-cigarettes in situations where smoking is not permitted from 2011 and 2020 in England?
2. How do dual users who report using e-cigarettes in situations where smoking is not permitted differ from those who use e-cigarettes for other reasons?
3. Has the sociodemographic or smoking profile of dual users who report using e-cigarettes in situations where smoking is not permitted changed between 2011 and 2020 and, if so, in what way?

## Materials and Methods

### Study Design and Population

Data were drawn from the ongoing Smoking Toolkit Study, a monthly cross-sectional survey of a representative sample of adults (≥16 years) in England designed to provide insights into population-wide influences on smoking and cessation by monitoring trends on a range of variables relating to smoking.^13^ The study uses a form of random location sampling to select a new sample of approximately 1700 adults aged at least 16 years each month. Participants complete a face-to-face computer-assisted survey with a trained interviewer. Comparisons with national data indicate that key variables such as sociodemographic characteristics and smoking prevalence are nationally representative.^13^

For the present study, we used data from respondents to the survey in the period from April 2011 (the first full quarter to include the items on e-cigarette use described below) through February 2020 (the most recent wave of data at the time of analysis). Our sample for analysis included respondents who, at the time of the survey, reported being a current cigarette smoker and current e-cigarette user (“dual users”).

### Measures

Smoking status was assessed with the question: “Which of the following best applies to you? (1) I smoke cigarettes (including hand rolled) every day; (2) I smoke cigarettes (including hand rolled), but not every day; (3) I do not smoke cigarettes at all, but I do smoke tobacco of some kind (eg, pipe, cigar, or shisha); (4) I have stopped smoking completely in the last year; (5) I stopped smoking completely more than a year ago; and (6) I have never been a smoker (ie, smoked for a year or more)” Participants who responded (1) or (2) were considered current cigarette smokers. Those who responded (3)–(6) were excluded from the analysis.

Current use of e-cigarettes was assessed with three questions: (1) “Which, if any, of the following are you currently using to help you cut down the amount you smoke?” (2) “Do you regularly use any of the following in situations when you are not allowed to smoke?” (3) “Are you using any of the following either to help you stop smoking, to help you cut down or for any other reason at all?” The list of response options varied across waves to capture the full range of alternative nicotine products available at the time of the survey but always included a response option for e-cigarettes. Participants who responded “electronic cigarette” to one or more of these questions were considered current e-cigarette users. Those who did not were excluded from the analysis. We have published widely on e-cigarette use using this measure (eg, refs ^14–16^), and it produces estimates of overall prevalence that align closely with surveys conducted by the Office for National Statistics^17^ and Action on Smoking and Health.^18^

Use of e-cigarettes for temporary abstinence in situations when smoking is not permitted was assessed with the question: “Do you regularly use any of the following in situations when you are not allowed to smoke?” Respondents who reported using e-cigarettes for this reason were coded 1 and those who did not were coded 0. Individual-level data were aggregated to produce quarterly population-level prevalence estimates for the trend analysis. For each quarter, the mean number of participants reporting use of e-cigarettes in situations when smoking is not permitted was divided by the mean sample size, multiplied by 100. For a few months (May, July, September, and November 2012, January and March 2013), data on e-cigarette use among smokers were not recorded. For these months, data were imputed using data from the previous and next month.

Sociodemographic characteristics assessed included age, sex, social grade (an occupational index of socioeconomic position, categorized as ABC1, which includes managerial, administrative, and professional and occupations, vs. C2DE, which includes semiroutine and routine occupations, manual occupations, never workers, and long-term unemployed^19^), and region (Government Office Region grouped into northern, central, and southern England). Ethnicity was not included because this information was not recorded before 2013.

Smoking characteristics assessed included number of cigarettes smoked per day, daily versus nondaily smoking, time to first cigarette (within 5 minutes vs. >5 minutes),^20^ strength of urges to smoke (an indicator of cigarette addiction),^21^ motivation to stop smoking,^22^ whether the participant was currently cutting down, current use of NRT, and whether the participant had tried to quit in the last year.

### Statistical Analysis

The analysis plan was preregistered on Open Science Framework (https://osf.io/5mu2r/). We made one amendment: we had intended to use data from April 2011 through March 2020 but data collection was not completed in March 2020 because of restrictions implemented to control the spread of COVID-19. Estimates for the first quarter of 2020 are thus based on data from January and February only.

#### Population-Level Trend Analysis

For analysis of prevalence trends, data were weighted using rim (marginal) weighting to match the English population profile on the dimensions of age, social grade, region, tenure, ethnicity, and working status within sex.^13,23–25^

We used generalized additive mixed models to regress time onto prevalence of e-cigarette use in situations where smoking is not permitted, applying a seasonal smoothing term. Generalized additive mixed models allow the incorporation of autocorrelation. The presence of autoregressive-1 autocorrelation [AR(1)] were assessed with the Durbin–Watson test and AR(2) and moving average −1 and −2 [MA(1) and MA(2)] autocorrelation with the autocorrelation function and the partial autocorrelation function. Higher order AR and MA terms were excluded as they were not believed to be plausible. The Durbin–Watson test was significant for all models except the cubic trend model, and the autocorrelation function and partial autocorrelation function indicated the presence of autocorrelation.

We assessed six trend models: (1) linear, (2) quadratic, (3) cubic, (4) power (log–log model), (5) exponential (log-level model), and (6) logarithmic (level-log model). Other functions (eg, quartic and quantic polynomial regressions) were not tested as we did not believe that they would reflect plausible underlying trends in prevalence and could lead to overfitting.

To identify the best overall models, all the resulting regression models were compared using the Akaike information criterion (AIC) as the primary measure of fit, and the adjusted *R*^2^ and Bayesian information criterion as secondary measures of fit. In general, the smaller the AIC and Bayesian information criterion, and larger the adjusted *R*^2^, the better the model fit. A prerequisite in using the AIC and Bayesian information criterion to compare models is that the dependent variable is on the same scale; thus, to ensure equivalence for the exponential trend and power trend models, a correction was applied to the AIC and Bayesian information criterion. This involved adding the Jacobian of the log transformation that is, $2\sum_{1}log\left(y_{i}\right)$ where *y* is the outcome variable of interest. The criteria for selecting the best fitting model was either the model with the lowest AIC or the simplest model if it was within two units of the model with the lowest AIC score. Model fit indices for all the models are shown in Supplementary Table 1.

Primary interpretation of the results was based on the best fitting model. The parameters relating to the linear and the best fitting models are reported in Results section. Results of standard regression models without adjustment for seasonality are shown in Supplementary Table 2. Orthogonal polynomials were used for model selection as they are uncorrelated but raw polynomials are reported for the final models.

#### Individual-Level Analyses

We used logistic regression on aggregated data across the entire study period to examine the extent to which sociodemographic and smoking characteristics were associated with use of e-cigarettes in situations where smoking is not permitted. Bivariate associations between use of e-cigarettes in situations where smoking is not permitted and each potential correlate were tested separately and independent associations were assessed with a multivariable model that included all variables. All analyses were performed on complete cases. The linearity assumption was met for each continuous variable.

In order to examine whether profiles of smokers who use e-cigarettes in situations where smoking is not permitted had changed over time, we used data collected in 2011–2012 (April 2011 to March 2012) and 2019–2020 (April 2019 to February 2020) to test the 2 × 2 interaction between survey year (2011–2012 vs. 2019–2020) and use of e-cigarettes in situations where smoking is not permitted (yes vs. no) for each sociodemographic and smoking-related characteristic. The reason the outcomes for these analyses were the sociodemographic or smoking-related characteristic of interest, rather than use of e-cigarettes in situations where smoking is not permitted (as in the previous analysis), is that these analyses were designed to test changes in the profile of smokers who use e-cigarettes in situations where smoking is not permitted, over and above changes in these characteristics that have occurred in the wide population of dual users of cigarettes and e-cigarettes (and not to simply describe changes in use of e-cigarettes in situations where smoking is not permitted within each subgroup). We used logistic regression for categorical outcomes and linear regression for continuous outcomes.

## Results

A total of 185 066 people responded to the survey from April 2011 through February 2020, of whom 5081 (2.7%, 95% CI = 2.7% to 2.8%) reported current use of both cigarettes and e-cigarettes (“dual users”). Complete data on sociodemographic and smoking characteristics were available for 4851 (95.5%) of participants (see Supplementary Table 3 for details of missing data).

Among dual users of cigarettes and e-cigarettes, the prevalence of use of e-cigarettes in situations where smoking is not permitted increased over the 9-year study period from 45.1% (95% CI = 25.6% to 67.2%; weighted *n* = 11.00/24.39) in the second quarter of 2011 to 70.5% (95% CI = 60.5% to 80.8%; weighted *n* = 59.55/84.47) in the first quarter of 2020.

**Figure 1.**
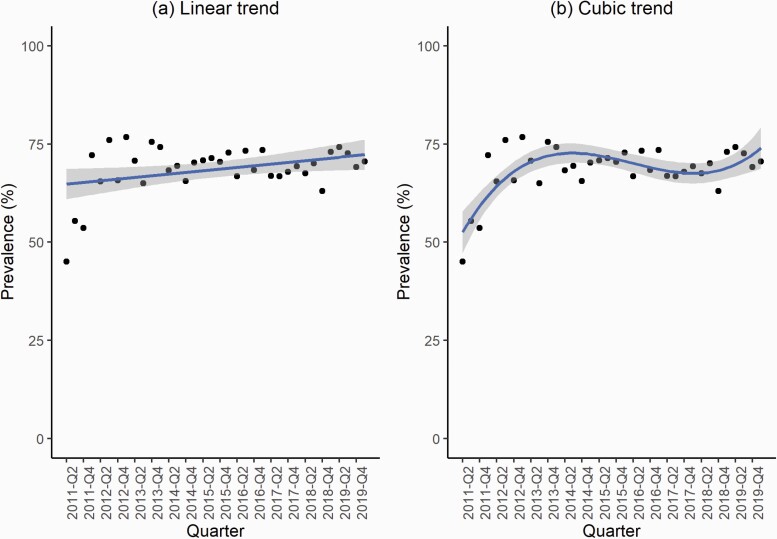
Raw and fitted prevalence of use of e-cigarettes in situations where smoking is not permitted from (a) linear and (b) best fitting models. Solid line, regression line; black dots, observed data; shaded area, 95% confidence interval of regression line.

The best fitting regression model was a cubic trend model without adjustment for autocorrelation (Figure 1, Table 1; model fit comparison Supplementary Table 1). This model indicated that among dual users of cigarettes and e-cigarettes, there was a decelerating increase in the prevalence of use of e-cigarettes in situations where smoking is not permitted between 2011 and 2014 (from an estimated 52.5% in Q2-2011 to 72.7% in Q3-2014), followed by a small decline between 2014 and 2018 (to 67.5% in Q1-2018), and a subsequent increase between 2018 and 2020 (to 74.0% in Q1-2020).

**Table 1.** Trends in Quarterly Prevalence of Use of E-Cigarettes in Situations Where Smoking Is not Permitted Among Dual Users of Cigarettes and E-Cigarettes in England, 2011–2020: Results of the Linear and Best Fitting Models

	*B*	95% CI		*p*
		Lower	Upper	
Linear model				
No autocorrelation				
*** *** Intercept	64.578	60.568	68.589	<.001
*** *** Time	0.214	0.025	0.403	.033
Autocorrelation				
*** *** Intercept	58.520	47.765	69.276	<.001
*** *** Time	0.428	−0.042	0.899	.084
Best fitting model (cubic model)				
No autocorrelation				
*** *** Intercept	48.606	42.085	55.127	<.001
*** *** Time	4.158	2.653	5.664	<.001
*** *** Time^2^	−0.224	−0.318	−0.130	<.001
*** *** Time^3^	0.004	0.002	0.005	<.001
Autocorrelation				
*** *** Intercept	49.192	43.265	55.119	<.001
*** *** Time	4.027	2.659	5.395	<.001
*** *** Time^2^	−0.216	−0.301	−0.131	<.001
*** *** Time^3^	0.003	0.002	0.005	<.001

All models were adjusted for seasonality. Linear model adjusted for autocorrelation included 2 autoregressive (AR) terms and 0 moving average (MA terms). Best fitting (cubic) model adjusted for autocorrelation included 1 AR term and 1 MA term. Intercept = value of the dependent variable at the start of the series. Time (linear model) = linear slope between time and the dependent variable. If the sign is positive then the dependent variable increases as time increases, if the sign is negative then the dependent variable decreases as time increases. Time (cubic trend model) = rate of change in the dependent variable at the start of the series. Time^2^ (cubic trend model) = the quadratic trend over the series. If the sign is positive then the model is convex (curvature is upward), if it is negative then the curve is concave (curvature is downward). Time^3^ (cubic trend model) = the cubic trend over the time series. If the sign is positive then the quadratic trend is increasingly positive as time increases, if it is negative then the quadratic trend is increasingly negative as time increases.

Table 2 summarizes associations between sociodemographic and smoking characteristics and use of e-cigarettes in situations where smoking is not permitted. There were significant independent associations with age, social grade, region, strength of urges to smoke, cutting down, current use of NRT, and past-year quit attempts. Those who were from more disadvantaged social grades (C2DE) reported stronger urges to smoke or had made a serious quit attempt in the past year had increased odds of reporting using an e-cigarette in situations where smoking is not permitted, and those who were aged at least 65 years (vs. 16–24 years), from the south of England (vs. the north), currently cutting down, or currently using NRT had decreased odds.

**Table 2.** Bivariate and Multivariable Logistic Regression Models of Associations Between Sociodemographic and Smoking Characteristics and Use of E-Cigarettes in Situations Where Smoking Is not Permitted Among Dual Users of Cigarettes and E-Cigarettes in England, 2011–2020

	*n*	%^a^	Bivariate		Multivariable	
			OR [95% CI]	*p*	OR_adj_ [95% CI]	*p*
Age in years						
*** *** 16–24	923	71.7	1.00	—	1.00	—
*** *** 25–34	1033	69.9	0.92 [0.75–1.11]	0.375	0.96 [0.78–1.17]	.653
*** *** 35–44	859	71.6	0.99 [0.81–1.22]	0.952	1.04 [0.84–1.29]	.740
*** *** 45–54	868	68.0	0.84 [0.68–1.02]	0.084	0.87 [0.70–1.08]	.199
*** *** 55–64	698	67.3	0.81 [0.66–1.01]	0.057	0.85 [0.68–1.07]	.161
*** *** ≥65	470	63.8	0.70 [0.55–0.88]	0.003	0.75 [0.59–0.97]	.025
Sex						
*** *** Men	2523	70.3	1.00	—	1.00	—
*** *** Women	2328	68.1	0.90 [0.80–1.02]	0.093	0.90 [0.79–1.02]	.097
Social grade						
*** *** ABC1	2176	66.6	1.00	—	1.00	—
*** *** C2DE	2675	71.4	1.25 [1.10–1.41]	<0.001	1.19 [1.05–1.36]	.008
Region						
*** *** North	1957	71.0	1.00	—	1.00	—
*** *** Central	1300	71.0	1.00 [0.86–1.17]	0.987	1.00 [0.85–1.17]	.975
*** *** South	1594	65.6	0.78 [0.68–0.90]	0.001	0.82 [0.71–0.95]	.008
Nondaily smoker						
*** *** No	4233	69.8	1.00	—	1.00	—
*** *** Yes	618	65.2	0.81 [0.68–0.97]	0.020	0.84 [0.69–1.03]	.087
Time to first cigarette						
*** *** >5 min	4010	68.4	1.00	—	1.00	—
*** *** Within 5 min	841	73.1	1.26 [1.06–1.48]	0.007	1.09 [0.91–1.31]	.368
High motivation to stop^c^						
*** *** No	3575	70.0	1.00	—	1.00	—
*** *** Yes	1276	67.1	0.87 [0.76–1.00]	0.052	1.00 [0.86–1.15]	.974
Currently cutting down						
*** *** No	1141	85.9	1.00	—	1.00	—
*** *** Yes	3710	64.1	0.29 [0.25–0.35]	<0.001	0.27 [0.23–0.33]	<.001
Current use of NRT						
*** *** No	4094	71.6	1.00	—	1.00	—
*** *** Yes	757	56.5	0.52 [0.44–0.61]	<0.001	0.49 [0.41–0.57]	<.001
Tried to quit in past year						
*** *** No	2344	68.9	1.00	—	1.00	—
*** *** Yes	2507	69.6	1.04 [0.92–1.17]	0.572	1.28 [0.12–1.46]	<.001
	***n***	**Mean (SD)** ^**b**^				
Cigarettes per day	4851	11.67 (8.28)/11.02 (7.64)	1.01 [1.00–1.02]	0.010	1.00 [0.99–1.01]	.911
Strength of urges to smoke^d^	4851	2.19 (1.06)/2.09 (1.03)	1.09 [1.03–1.16]	0.003	1.11 [1.04–1.19]	.003

OR = odds ratio; CI = confidence interval; SD = standard deviation; NRT = nicotine replacement therapy.

^a^Percentage of current smokers in each category who reported use of e-cigarettes in situations where smoking is not permitted.

^b^Mean (SD) for the group of current smokers who reported/did not report use of e-cigarettes in situations where smoking is not permitted.

^c^Defined as really wanting to stop smoking within the next 3 months.

^d^Self-rated strength of urges to smoke over the past 24 hours, on a scale from 0 (not at all) to 5 (extremely strong).

Table 3 summarizes the sociodemographic and smoking characteristics of dual users of cigarettes and e-cigarettes who did versus did not report using e-cigarettes in situations where smoking is not permitted in 2011–2012 and 2019–2020. There was no significant interaction between survey year (2011–2012 vs. 2019–2020) and use of e-cigarettes in situations where smoking is not permitted (yes vs. no) for any characteristic.

**Table 3.** Sociodemographic and Smoking Profile of Dual Users of Cigarettes and E-Cigarettes in England Who Use E-Cigarettes in Situations Where Smoking Is not Permitted: 2011–2012 and 2019–2020

	Use of e-cigarettes in situations where smoking is not permitted						Interaction^a^	
	Yes^b^			No^c^				
	2011–2012	2019–2020	Change^d^	2011–2012	2019–2020	Change^d^	OR [95% CI]	*p*
*n*	95	337	—	60	139	—	—	—
Age in years (%)								
*** *** 16–24	18.9	19.9	1.0	10.0	26.6	16.6	0.51 [0.12–2.14]	.354
*** *** 25–34	12.6	24.0	11.4	18.3	20.9	2.6	2.15 [0.54–8.55]	.278
*** *** 35–44	24.2	15.1	−9.1	15.0	13.7	−1.3	0.88 [0.22–3.54]	.858
*** *** 45–54	14.7	16.3	1.6	25.0	10.8	−14.2	3.30 [0.83–13.15]	.091
*** *** 55–64	15.8	15.4	−0.4	16.7	15.1	−1.6	1.39 [0.34–5.61]	.648
*** *** ≥65	13.7	9.2	−4.5	15.0	12.9	−2.1	Ref	—
Women (%)	44.2	45.4	1.2	46.7	42.4	−4.3	1.25 [0.58–2.67]	.573
Social grade C2DE (%)	57.9	48.4	−9.5	48.3	46.0	−2.3	0.75 [0.35–1.60]	.452
Region (%)								
*** *** North	36.8	37.4	0.6	30.0	32.4	2.4	0.95 [0.38–2.39]	.954
*** *** Central	28.4	29.7	1.3	33.3	32.4	−0.9	1.09 [0.43–2.76]	.856
*** *** South	34.7	32.9	−1.8	36.7	35.3	−1.4	Ref	—
Nondaily smoker (%)	7.4	14.5	7.1	8.3	20.9	12.6	0.74 [0.20–2.71]	.646
First cigarette within 5 min of waking (%)	20.0	13.6	−6.4	16.7	13.7	−3.0	0.80 [0.29–2.22]	.666
High motivation to stop (%)^e^	31.6	24.0	−7.6	30.0	29.5	−0.5	0.70 [0.31–1.61]	.403
Currently cutting down (%)	71.6	69.7	−1.9	93.3	84.9	−8.4	2.28 [0.67–7.75]	.187
Currently using NRT (%)	17.9	7.1	−10.8	28.3	22.3	−6.0	0.49 [0.19–1.27]	.139
Tried to quit in past year (%)	62.1	47.8	−14.3	55.0	46.8	−8.2	0.78 [0.36–1.67]	.518
							**Beta [95% CI]**	***p***
Cigarettes per day (mean [SD])	14.91 (9.33)	9.57 (7.42)	−5.34	12.70 (8.44)	8.84 (6.66)	−3.86	−1.48 [−4.40 to 1.43]	.318
Strength of urges to smoke (mean [SD])^f^	2.51 (1.01)	2.00 (0.99)	−0.51	2.13 (0.96)	1.82 (1.05)	−0.31	−0.19 [−0.57 to 0.19]	.330

OR = odds ratio; CI = confidence interval; SD = standard deviation; NRT = nicotine replacement therapy; ref = referent category.

^a^The 2 × 2 interaction between survey year (2011–2012 vs. 2019–2020) and use of e-cigarettes in situations where smoking is not permitted (yes vs. no) for each sociodemographic and smoking-related characteristic.

^b^Descriptive characteristics of dual users responding to the Smoking Toolkit Study surveys in April 2011 to March 2012 and April 2019 to February 2020 (aggregated monthly data) who use e-cigarettes in situations where smoking is not permitted.

^c^Descriptive characteristics of dual users responding to the Smoking Toolkit Study surveys in April 2011 to March 2012 and April 2019 to February 2020 (aggregated monthly data) who do not use e-cigarettes in situations where smoking is not permitted.

^d^Percent or mean change between 2011–2012 and 2019–2020.

^e^Defined as really wanting to stop smoking within the next 3 months.

^f^Self-rated strength of urges to smoke over the past 24 hours, on a scale from 0 (not at all) to 5 (extremely strong).

## Discussion

Between 2011 and 2020, the prevalence of use of e-cigarettes in situations where smoking is not permitted by dual users of cigarettes and e-cigarettes in England followed a cubic trend, increasing from 2011 to 2014, decreasing slightly from 2014 to 2018, then increasing from 2018 to 2020. Across the entire 9-year study period, odds of use of e-cigarettes in situations where smoking is not permitted were higher among dual users who were more socioeconomically disadvantaged, those who were more addicted, and those who had tried to quit in the past year. However, odds of use of e-cigarettes in situations where smoking is not permitted were lower among older smokers, those who lived in the south of England, those who were currently cutting down, and those who were currently using NRT. The profile of dual users who used e-cigarettes in situations where smoking is not permitted was stable over time, with no changes in the sociodemographic or smoking characteristics of this population between 2011–2012 and 2019–2020 over and above changes observed in dual users who used e-cigarettes for other reasons.

Prevalence of use of e-cigarettes in situations where smoking is not permitted among dual users of cigarettes and e-cigarettes has broadly followed the same pattern as prevalence of e-cigarette use among adults in England. As e-cigarettes rapidly became popular between 2011 and 2014, a growing proportion of dual users reported using them in situations where smoking is not permitted. As the prevalence of e-cigarette use plateaued from 2014 onward, the prevalence of use in situations where smoking is not permitted did not change substantially. With businesses and public transport operators increasingly extending smoke-free policies to include vaping, we had anticipated that use of e-cigarettes in situations where smoking is not permitted might have declined over recent years. However, we did not find this to be the case; instead the best fitting model suggested a small increase between 2018 and 2020. Stealth vaping is the practice of vaping discreetly where e-cigarette use is prohibited. It is widely discussed online and appears common among experienced users in the United States.^26,27^ An increase in stealth vaping may partly account for the apparent discrepancy between the prevalence of the use of e-cigarettes where smoking is not permitted and the increasingly extensive policies to include vaping in smoke-free policies. It is also possible that users are doing less vaping in situations where smoking is not permitted—but continue to do at least some; our study did not consider the frequency with which—or locations—an individual used an e-cigarette where smoking is not permitted.

The characteristics of dual users who reported using e-cigarettes in situations where smoking is not permitted are those typically associated with higher smoking prevalence (ie, socioeconomic disadvantage, younger age, and living in the North of England^28,29^) and dependence (ie, socioeconomic disadvantage, failed quit attempts, and not cutting down^30,31^). The association with failed quit attempts could also indicate greater motivation to quit in this group, although there was no significant association with our more direct assessment of motivation to stop smoking. Despite a substantial increase in the prevalence of use of e-cigarettes in situations where smoking is not permitted over the study period, there was no substantial change in the profile of dual users of cigarettes and e-cigarettes who did so relative to dual users who did not use e-cigarettes in situations where smoking is not permitted.

These findings have implications for regulation of the use of e-cigarettes in public places. Assuming that of the 44.6 million adults (≥16 years) in England,^28^ 2.7% are dual users of cigarettes and e-cigarettes and 70.5% (Q1-2020) of these people use e-cigarettes in situations where smoking is not permitted, any restrictions on the use of e-cigarettes in public places could affect in the region of 850 000 smokers in England. The ability to use nicotine in smoke-free settings appears to be an important part of the appeal of e-cigarettes.^3,4,6^ Our results suggest that while dual users are currently prohibited from vaping in some public places, it has not substantially reduced all use in public settings. However, it is possible that if e-cigarette use was banned completely in public places, smokers may be deterred from initiating or continuing use of e-cigarettes alongside combustible tobacco. This could undermine quitting: A recent prospective study found that smokers who also used e-cigarettes were slightly more likely to make a serious attempt to quit than those who exclusively smoked.^14^ The association we observed with social grade means disadvantaged smokers would be disproportionately affected, suggesting such restrictions may contribute to inequalities in smoking and associated health outcomes.^30^ In making decisions on the regulation of use of e-cigarettes in public places, there is thus a need to weigh what appear to be negligible risks to bystanders (ie, what currently appears to be no evidence of health risks associated with inhaling second-hand vapor^5^) against the risk of deterring uptake of e-cigarette use as a potential route to quitting and the implications of this for population health.

Strengths of this study include the large, representative sample and monthly data collection allowing examination of trends with greater granularity than would be achieved in other surveys, which collect data annually. However, there were limitations. The measure of use of e-cigarettes in situations where smoking is not permitted was not context specific, so we were not able to distinguish between use in public places with blanket bans (eg, restaurants, train stations) and use at home (eg, where landlords or other family members prohibit smoking). In addition, the relatively small proportion of the population who reported dual use of cigarettes and e-cigarettes meant we lacked sufficient data to analyze monthly trends, but we were able to aggregate data quarterly and account for seasonal variation in use of e-cigarettes in situations where smoking is not permitted.

In conclusion, use of e-cigarettes in situations where smoking is not permitted is common among dual cigarette and e-cigarette users in England and has increased nonlinearly since 2011. Profiles of those who do so have remained stable over time, with dual users who were younger, more disadvantaged, more addicted, had failed to quit in the last year, and were not attempting to cut down more likely to use e-cigarettes in situations where smoking is not permitted.

## Supplementary Material

A Contributorship Form detailing each author’s specific involvement with this content, as well as any supplementary data, are available online at https://academic.oup.com/ntr.

ntab119_suppl_Supplementary_Table_S1-S3

ntab119_suppl_Supplementary_Taxonomy-form

## Data Availability

The datasets used and analyzed during the current study are available from the corresponding author on request.

## References

[1] West R , Proudfoot H, Beard E, Brown J. *Electronic Cigarettes in England—Latest Trends [Internet]*. Smoking in England; 2019 [cited May 26, 2019]. http://www.smokinginengland.info/latest-statistics/. Accessed February 1, 2021.

[2] Office for National Statistics. *Adult Smoking Habits in the UK: 2019 [Internet]*; 2020. https://www.ons.gov.uk/peoplepopulationandcommunity/healthandsocialcare/healthandlifeexpectancies/bulletins/adultsmokinghabitsingreatbritain/2019#the-proportion-who-are-current-smokers-in-the-uk-its-consistent-countries-and-local-areas-2011-to-2019. Accessed February 1, 2021.

[3] Simonavicius E , McNeill A, Arnott D, Brose LS. What factors are associated with current smokers using or stopping e-cigarette use? Drug Alcohol Depend. 2017;173:139–143. 28246049 10.1016/j.drugalcdep.2017.01.002 PMC5380653

[4] Patel D , Davis KC, Cox S, et al. Reasons for current e-cigarette use among U.S. adults. Prev Med. 2016;93:14–20. 27612572 10.1016/j.ypmed.2016.09.011 PMC5316292

[5] McNeill A , Brose LS, Calder R, Bauld L, Robson D. Evidence review of e-cigarettes and heated tobacco products 2018. A report commissioned by Public Health England [Internet]; 2018 [cited June 21, 2018]. https://www.gov.uk/government/publications/e-cigarettes-and-heated-tobacco-products-evidence-review/evidence-review-of-e-cigarettes-and-heated-tobacco-products-2018-executive-summary. Accessed February 1, 2021.

[6] Biener L , Hargraves JL. A longitudinal study of electronic cigarette use among a population-based sample of adult smokers: association with smoking cessation and motivation to quit. Nicotine Tob Res. 2015;17(2):127–133. 25301815 10.1093/ntr/ntu200 PMC4375383

[7] Rutten LJ , Blake KD, Agunwamba AA, et al. Use of e-cigarettes among current smokers: associations among reasons for use, quit intentions, and current tobacco use. Nicotine Tob Res. 2015;17(10):1228–1234. 25589678 10.1093/ntr/ntv003 PMC4592339

[8] Science and Technology Committee. E-Cigarettes [Internet]. London, UK: House of Commons; 2018 [cited September 3, 2019]. https://publications.parliament.uk/pa/cm201719/cmselect/cmsctech/505/50502.htm. Accessed February 1, 2021.

[9] World Health Organization. WHO Report on the Global Tobacco Epidemic, 2019 [Internet]. Geneva, Switzerland: World Health Organization; 2019 [cited March 9, 2019]. https://apps.who.int/iris/bitstream/handle/10665/326043/9789241516204-eng.pdf?ua=1. Accessed February 1, 2021.

[10] Public Health England. *Use of E-Cigarettes in Public Places and Workplaces: Advice to Inform Evidence-Based Policy-Making [Internet]*; 2016 [cited September 3, 2019]. https://www.gov.uk/government/publications/use-of-e-cigarettes-in-public-places-and-workplaces. Accessed February 1, 2021.

[11] Beard E , McNeill A, Aveyard P, Fidler J, Michie S, West R. Association between use of nicotine replacement therapy for harm reduction and smoking cessation: a prospective study of English smokers. Tob Control. 2013;22(2):118–122. 22135165 10.1136/tobaccocontrol-2011-050007

[12] West R , Brown J, Shahab L. *Written Evidence Submitted by University College London, Tobacco and Alcohol Research Group (UTARG) (ECG0047) [Internet]*; 2017 [cited October 11, 2018]. http://data.parliament.uk/writtenevidence/committeeevidence.svc/evidencedocument/science-and-technology-committee/ecigarettes/written/75276.html. Accessed February 1, 2021.

[13] Fidler JA , Shahab L, West O, et al. ‘The Smoking Toolkit Study’: a national study of smoking and smoking cessation in England. BMC Public Health. 2011;11:479. 21682915 10.1186/1471-2458-11-479 PMC3145589

[14] Jackson SE , Shahab L, West R, Brown J. Associations between dual use of e-cigarettes and smoking cessation: a prospective study of smokers in England. Addict Behav. 2020;103:106230. 31841827 10.1016/j.addbeh.2019.106230 PMC6970222

[15] Jackson SE , Kotz D, West R, Brown J. Moderators of real-world effectiveness of smoking cessation aids: a population study. Addiction. 2019;114(9):1627–1638. 31117151 10.1111/add.14656 PMC6684357

[16] Beard E , West R, Michie S, Brown J. Association between electronic cigarette use and changes in quit attempts, success of quit attempts, use of smoking cessation pharmacotherapy, and use of stop smoking services in England: time series analysis of population trends. BMJ. 2016;354:i4645. 27624188 10.1136/bmj.i4645

[17] Office for National Statistics. *E-Cigarette Use in Great Britain [Internet]*; 2020 [cited April 12, 2021]. https://www.ons.gov.uk/peoplepopulationandcommunity/healthandsocialcare/drugusealcoholandsmoking/datasets/ecigaretteuseingreatbritain. Accessed February 1, 2021.

[18] Action on Smoking and Health. Use of E-Cigarettes Among Adults in Great Britain, 2020 [Internet]. Action on Smoking and Health; 2020 [cited April 12, 2021]. https://ash.org.uk/information-and-resources/fact-sheets/statistical/use-of-e-cigarettes-among-adults-in-great-britain-2020/. Accessed February 1, 2021.

[19] National Readership Survey. *Social Grade—Definitions and Discriminatory Power [Internet]*; 2007 [cited October 1, 2012]. http://www.nrs.co.uk/lifestyle.html. Accessed February 1, 2021.

[20] Heatherton TF , Kozlowski LT, Frecker RC, Fagerström KO. The Fagerström Test for Nicotine Dependence: a revision of the Fagerström Tolerance Questionnaire. Br J Addict. 1991;86(9):1119–1127. 1932883 10.1111/j.1360-0443.1991.tb01879.x

[21] Fidler JA , Shahab L, West R. Strength of urges to smoke as a measure of severity of cigarette dependence: comparison with the Fagerström Test for Nicotine Dependence and its components. Addiction. 2011;106(3):631–638. 21134020 10.1111/j.1360-0443.2010.03226.x

[22] Kotz D , Brown J, West R. Predictive validity of the Motivation To Stop Scale (MTSS): a single-item measure of motivation to stop smoking. Drug Alcohol Depend. 2013;128(1–2):15–19. 22943961 10.1016/j.drugalcdep.2012.07.012

[23] Beard E , Brown J, West R, et al. Protocol for a national monthly survey of alcohol use in England with 6-month follow-up: ‘the Alcohol Toolkit Study’. BMC Public Health. 2015;15:230. 25884652 10.1186/s12889-015-1542-7 PMC4363185

[24] Kock L , Shahab L, Moore G, et al. Protocol for expansion of an existing national monthly survey of smoking behaviour and alcohol use in England to Scotland and Wales: the Smoking and Alcohol Toolkit Study. Wellcome Open Res. 2021;6:67. 34458587 10.12688/wellcomeopenres.16700.1 PMC8370132

[25] Brown J , West R, Angus C, et al. Comparison of brief interventions in primary care on smoking and excessive alcohol consumption: a population survey in England. Br J Gen Pract. 2016;66(642):e1–e9. 26719481 10.3399/bjgp16X683149 PMC4684029

[26] Vaping360. Stealth and Zero Vaping: What It Is and How to Do It [Internet]. Vaping360; 2018 [cited June 16, 2020]. https://vaping360.com/learn/stealth-vaping/. Accessed February 1, 2021.

[27] Yingst JM , Lester C, Veldheer S, Allen SI, Du P, Foulds J. E-cigarette users commonly stealth vape in places where e-cigarette use is prohibited. Tob Control. 2019;28(5):493–497. 30097510 10.1136/tobaccocontrol-2018-054432 PMC9703983

[28] Office for National Statistics. *Adult Smoking Habits in England [Internet]*; 2019 [cited April 17, 2020]. https://www.ons.gov.uk/peoplepopulationandcommunity/healthandsocialcare/healthandlifeexpectancies/datasets/adultsmokinghabitsinengland. Accessed February 1, 2021.

[29] Beard E , Brown J, West R, Angus C, Kaner E, Michie S. Healthier central England or North-South divide? Analysis of national survey data on smoking and high-risk drinking. BMJ Open. 2017;7(3):e014210. 10.1136/bmjopen-2016-014210 PMC5353327 28249851

[30] Hiscock R , Bauld L, Amos A, Fidler JA, Munafò M. Socioeconomic status and smoking: a review. Ann N Y Acad Sci. 2012;1248:107–123. 22092035 10.1111/j.1749-6632.2011.06202.x

[31] Zhou X , Nonnemaker J, Sherrill B, Gilsenan AW, Coste F, West R. Attempts to quit smoking and relapse: factors associated with success or failure from the ATTEMPT cohort study. Addict Behav. 2009;34(4):365–373. 19097706 10.1016/j.addbeh.2008.11.013

