# Supplementary material for: Smokers’ Use of E-Cigarettes in Situations Where Smoking Is not Permitted in England: Quarterly Trends 2011–2020 and Associations With Sociodemographic and Smoking Characteristics
Source: Nicotine Tob Res. 2021 Jun 5;23(11):1831–8. doi: 10.1093/ntr/ntab119 (PMC8496468; doi:10.1093/ntr/ntab119)
Supplement: ntab119_suppl_Supplementary_Table_S1-S3 [file ntab119_suppl_supplementary_table_s1-s3.docx]

| **Supplementary Table 1** Indices of fit for the regression models | | | | |
| --- | --- | --- | --- | --- |
|  | | **AIC** | **BIC** | **Adj. R^2^** |
| **No autocorrelation** | |  |  |  |
|  | Linear trend model | 235.2 | 240.0 | 0.10 |
|  | Quadratic trend model | 229.6 | 235.9 | 0.25 |
|  | Cubic trend model | 215.9 | 223.8 | 0.50 |
|  | Logarithmic trend model | 227.0 | 231.7 | 0.29 |
|  | Exponential trend model | 242.2 | 246.9 | 0.12 |
|  | Power trend model | 232.8 | 237.6 | 0.32 |
| **Autocorrelation** | |  |  |  |
|  | Linear trend model | 230.2 | 239.7 | 0.03 |
|  | Quadratic trend model | 227.0 | 238.1 | 0.15 |
|  | Cubic trend model | 217.5 | 230.2 | 0.50 |
|  | Logarithmic trend model | 223.0 | 232.5 | 0.10 |
|  | Exponential trend model | 234.7 | 244.2 | 0.09 |
|  | Power trend model | 225.6 | 235.1 | 0.09 |
| Note: Shading indicates selected model out of all possible models. All models are adjusted for seasonality. | | | | |

| **Supplementary Table 2** Results of the linear and best fitting standard regression models | | | | | |  |
| --- | --- | --- | --- | --- | --- | --- |
|  | |  | **95% CI** | |  | |
|  | | ***B*** | **lower** | **upper** | ***p*** | |
| **Linear model** | |  |  |  |  | |
|  | Intercept | 64.578 | 60.420 | 68.736 | <0.001 | |
|  | Time | 0.214 | 0.018 | 0.410 | 0.033 | |
| **Best fitting model (cubic model)** | |  |  |  |  | |
|  | Intercept | 48.606 | 41.829 | 55.383 | <0.001 | |
|  | Time | 4.158 | 2.594 | 5.723 | <0.001 | |
|  | Time^2^ | -0.224 | -0.322 | -0.126 | <0.001 | |
|  | Time^3^ | 0.004 | 0.002 | 0.005 | <0.001 | |
| Note: Intercept = value of the dependent variable at the start of the series.  Time (linear model) = linear slope between time and the dependent variable. If the sign is positive then the dependent variable increases as time increases, if the sign is negative then the dependent variable decreases as time increases.  Time (cubic trend model) = rate of change in the dependent variable at the start of the series.  Time^2^ (cubic trend model) = the quadratic trend over the series. If the sign is positive then the model is convex (curvature is upwards), if it is negative then the curve is concave (curvature is downwards).  Time^3^ (cubic trend model) = the cubic trend over the time series. If the sign is positive then the quadratic trend is increasingly positive as time increases, if it is negative then the quadratic trend is increasingly negative as time increases. | | | | | |  |

| **Supplementary Table 3** Missing data | |
| --- | --- |
|  | ***n* (%) missing** |
| Age | 0 (0) |
| Sex | 0 (0) |
| Social grade | 0 (0) |
| Region | 6 (0.1) |
| Non-daily smoker | 0 (0) |
| Time to first cigarette | 18 (0.4) |
| High motivation to stop | 7 (0.1) |
| Currently cutting down | 3 (0.1) |
| Current use of NRT | 0 (0) |
| Tried to quit in past year | 120 (2.4) |
| Cigarettes per day | 83 (1.6) |
| Strength of urges to smoke | 18 (0.4) |
|  | |
